# Supplementary material for: Arc regulates a second-guessing cognitive bias during naturalistic foraging through effects on discrete behavior modules
Source: iScience. 2023 Apr 27;26(5):106761. doi: 10.1016/j.isci.2023.106761 (PMC10196573; doi:10.1016/j.isci.2023.106761)
Supplement: Document S1. Figures S1–S4 [file mmc1.pdf]

iScience, Volume *26*

Supplemental information

Arc regulates a second-guessing cognitive bias during naturalistic foraging through effects on discrete behavior modules

Alicia Ravens, Cornelia N. Stacher-Hörndli, Jared Emery, Susan Steinwand, Jason D. Shepherd, and Christopher Gregg

SUPPLEMENTAL FIGURES AND LEGENDS

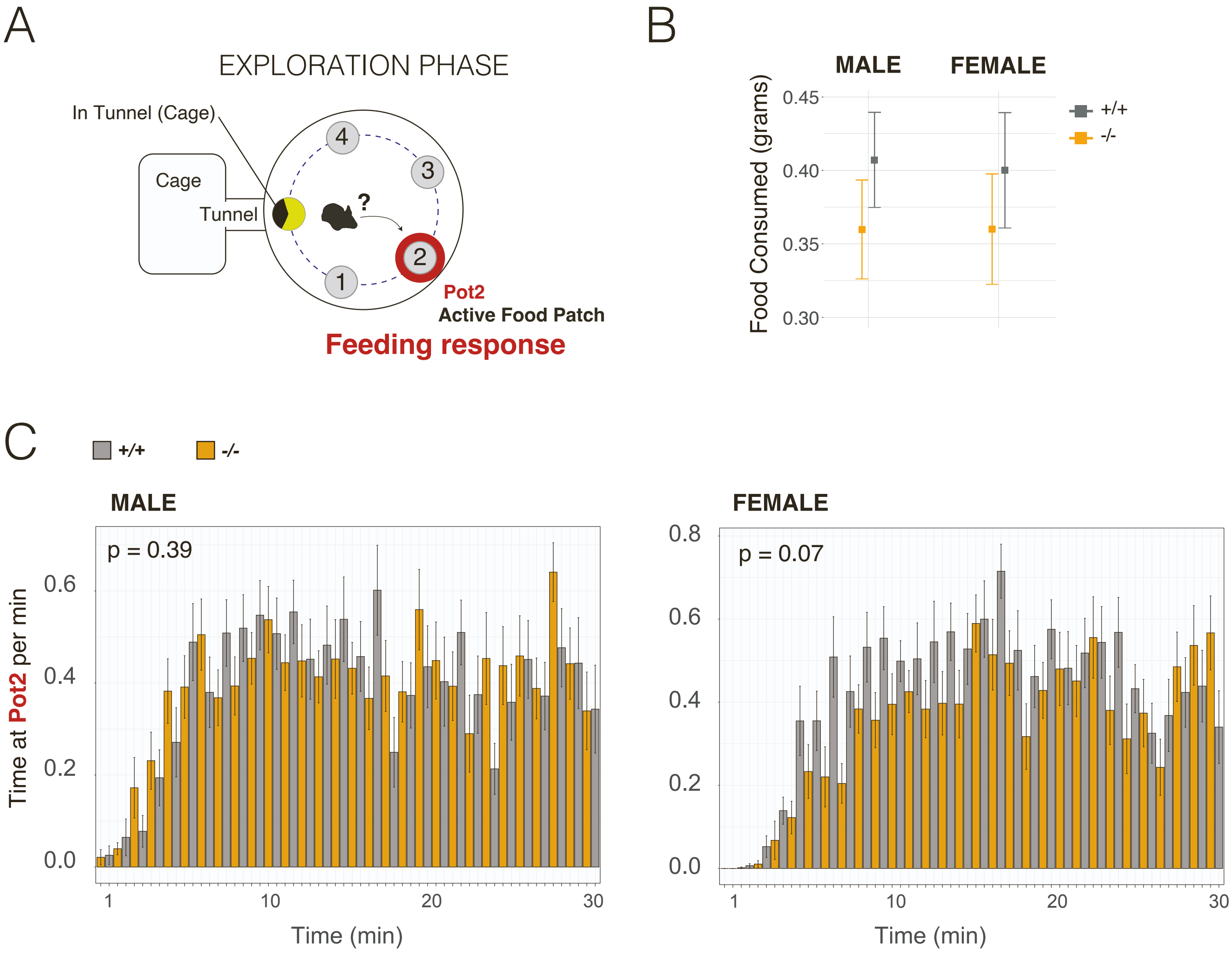
A
EXPLORATION PHASE
In Tunnel (Cage)
Cage
Tunnel
4
3
2
1
?
Pot2
Active Food Patch
Feeding response
B
MALE
FEMALE
Food Consumed (grams)
0.45
0.40
0.35
0.30
+/+
-/-
C
+/+
-/-
MALE
FEMALE
p = 0.39
p = 0.07
Time at Pot2 per min
0.0
0.2
0.4
0.6
0.8
1
10
20
30
Time (min)

FIGURE S1. Related to Figure 2. *Arc*$^{-/-}$ mice do not show significant changes to food intake or interactions with the food patch (Pot2) during the naïve Exploration phase.

(**A**) Schematic summary of the Exploration phase.

(**B**) The plot shows the total food consumed by *Arc*$^{-/-}$ (orange) versus $^{+/+}$ (black) mice in the Exploration phase. A significant difference is not observed. N=15, One-way Anova.

(**C**) The plot shows total time at the food patch during the Exploration phase (Pot2) broken down by 1 minute time bins. A significant difference is not observed between *Arc*$^{-/-}$ and $^{+/+}$ mice for males or females. N=15, mean+SEM, KS test comparison of distributions.

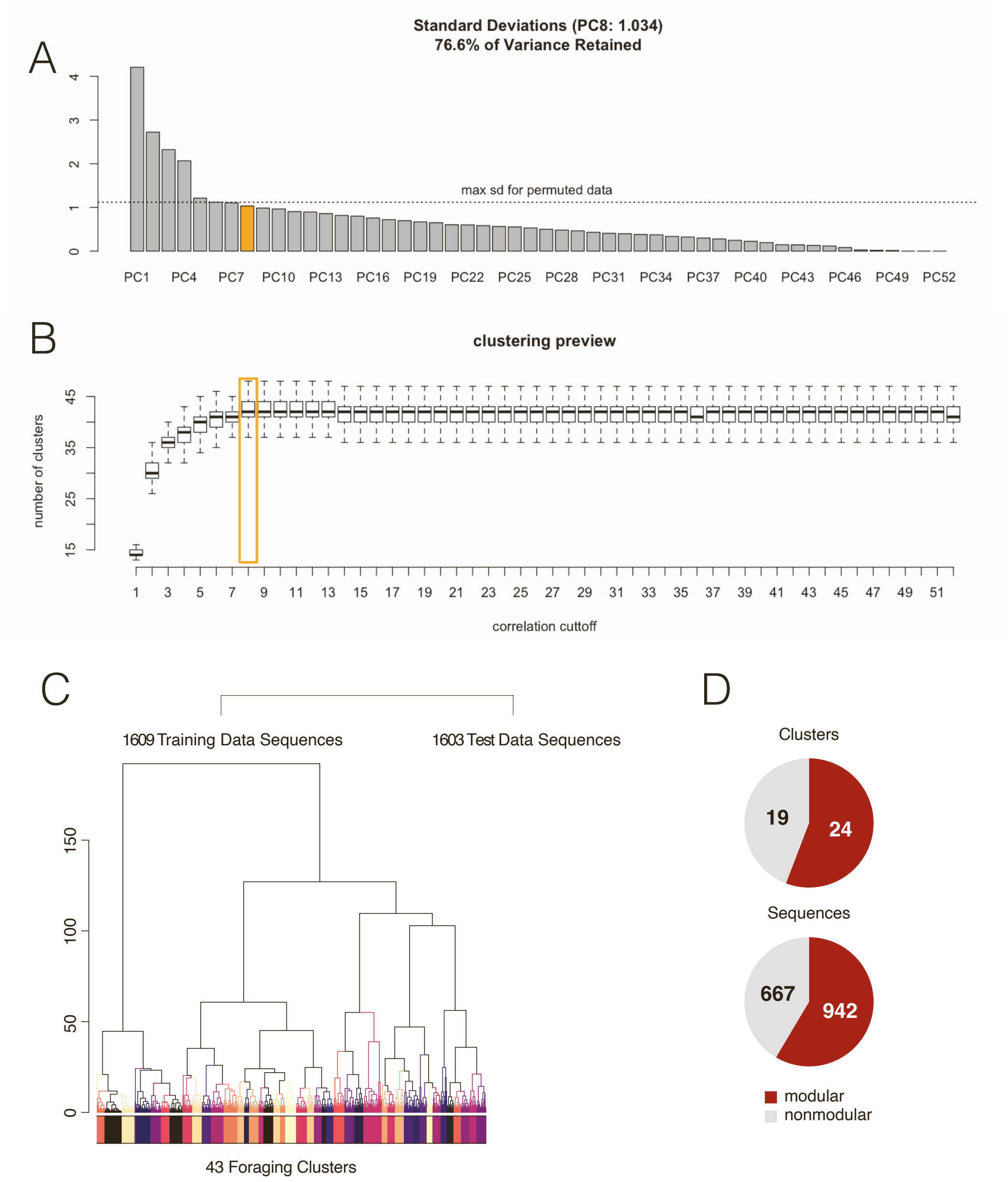
A
Standard Deviations (PC8: 1.034)
76.6% of Variance Retained
max sd for permuted data
PC1 PC4 PC7 PC10 PC13 PC16 PC19 PC22 PC25 PC28 PC31 PC34 PC37 PC40 PC43 PC46 PC49 PC52
B
clustering preview
number of clusters
correlation cuttoff
C
1609 Training Data Sequences
1603 Test Data Sequences
43 Foraging Clusters
D
Clusters
19
24
Sequences
667
942
modular
nonmodular

FIGURE S2. Identification of foraging modules. Related to Figure 4.

(**A**) The plot shows the number of principle components (PCs) and proportion of variance explained by each PC. The chosen PC cutoff is in orange at the elbow of the plot and captures 76% of variance in the data for hundreds of measures describing discrete round trip foraging excursions from the home.

(**B**) The plot shows the number of clusters discovered with the selected PCs based on different cutoffs for the number of PCs chosen. The data reveal that the maximum cluster number and minimum standard deviation in terms of cluster number is achieved using 8 PCs.

(**C**) Shows the DeepFeats training and testing partitions and clustering results to identify behavioral modules. Dynamic tree cutting yielded 43 candidate clusters in the training data for module testing.

(**D**) The pie charts show the number of significant modules (red) versus training set clusters that are not modular (grey) (ie. Significantly reproducible in the test data). The number of round-trip excursions found to be modular (red) versus non-modular (grey) is shown below. In-group proportion permutation test, Q-value <0.1.

(**E** and **F**) The plots show representative X-Y coordinate traces of the mouse center point body movement pattern over time (Z-axis) for examples of the decision and action sequences for significant behavior modules, including two modules involving increased interactions with the food patch Pot4 (4) in the Foraging phase (E, Modules 1 and 12), compared to a module that involves increased interaction with the former food patch Pot2 (2) (F, Module 21). The traces are for the excursions in the data that are closest to the centroid of the cluster testing significant for modularity. Traces are colored by

movement velocity (Black is slow or stopped and pink is moving). Mouse shadows illustrate the behavior. The dark circle is the tunnel connecting the home (H) and arena.

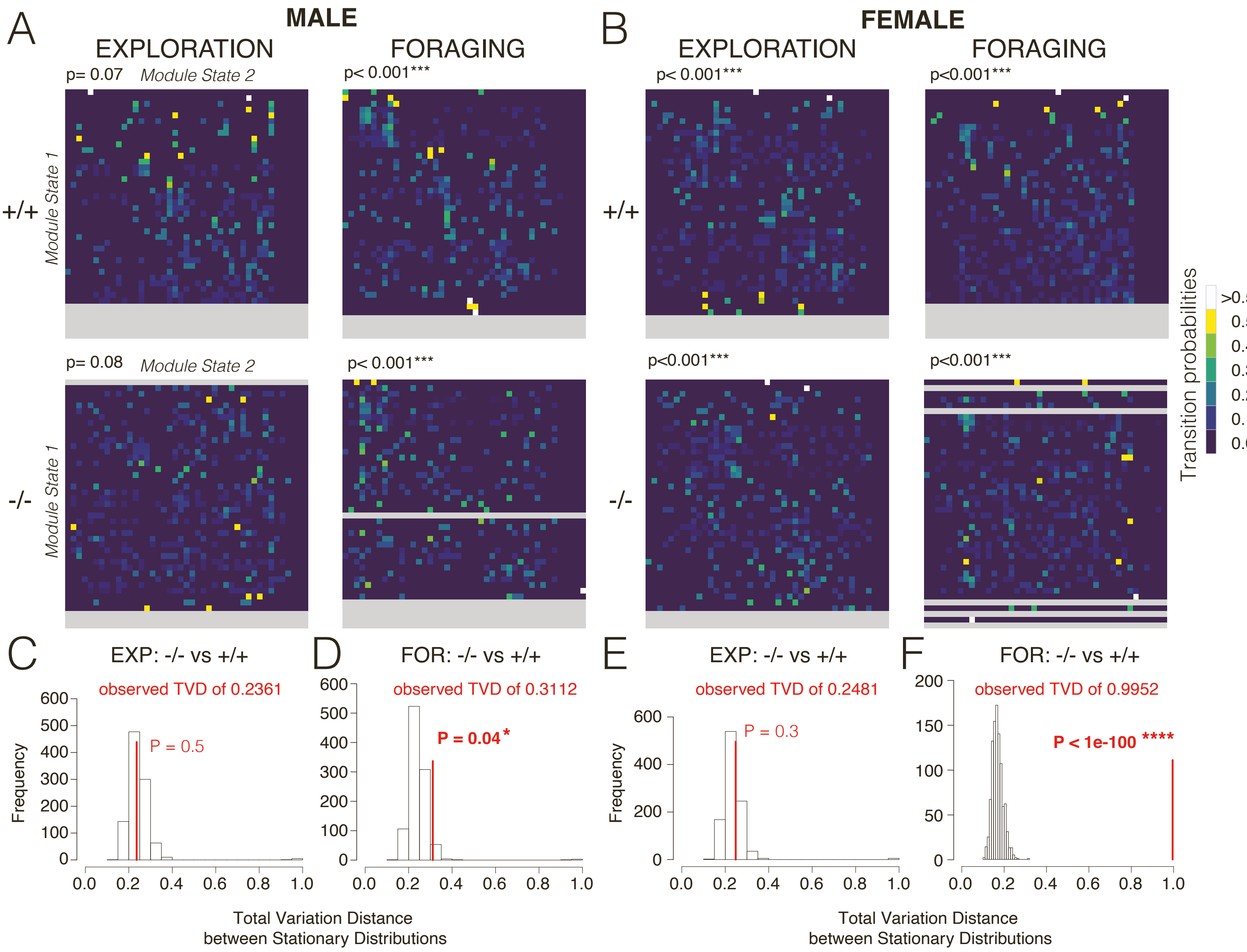

A
MALE
EXPLORATION
FORAGING
B
FEMALE
EXPLORATION
FORAGING
p= 0.07
Module State 2
p< 0.001***
p< 0.001***
p<0.001***
Module State 1
+/+
p= 0.08
Module State 2
p< 0.001***
p<0.001***
p<0.001***
-/-
Transition probabilities
>0.5
0.5
0.4
0.3
0.2
0.1
0.0
C
EXP: -/- vs +/+
observed TVD of 0.2361
P = 0.5
D
FOR: -/- vs +/+
observed TVD of 0.3112
P = 0.04*
E
EXP: -/- vs +/+
observed TVD of 0.2481
P = 0.3
F
FOR: -/- vs +/+
observed TVD of 0.9952
P < 1e-100 ****
Frequency
Total Variation Distance
between Stationary Distributions

FIGURE S3. Loss of *Arc* significantly changes the sequential order of module expression in the Foraging phase. Related to Figure 4.

(**A and B**) The heatmaps show transition matrices for module expression sequences during foraging behaviors in the Exploration and Foraging phase in males (A) and females (B). The y-axis shows Module State 1 and x-axis is Module State 2, where the intersections are the transition frequencies in a heatmap that shows module-to-module transition frequencies (see legend). Module expression transitions are significantly stereotyped and non-random in the Exploration and Foraging phases in *Arc*$^{-/-}$ and +/+ mice, which is statistically shown by a Fisher's Exact Test of independence between the rows (Module State 1) and the columns (Module State 2). The p-value shown above matrix and a significant effect indicates dependence, which is observed in all cases, though only a trend is observed in males during Exploration phase. Grey rows are modules that are not expressed due to being specific to either the Exploration or Foraging phases, or not expressed by a particular genotype. (n=15)

(**C-F**) The plots show results of a permutation test comparing the transition matrices between Arc +/+ versus -/- mice in the Exploration (EXP, C and E) and Foraging (FOR, D and F) phases for males (C and D) and females (D and F). The permutation test compares the stationary probability distributions of the transition matrices for *Arc*$^{-/-}$ and $^{+/+}$ mice. The results show a significant difference in module expression transitions between *Arc*$^{-/-}$ and $^{+/+}$ mice specifically in the Foraging phase in males and females. Thus, *Arc* affects the sequential order of module expression in $^{-/-}$ versus $^{+/+}$ mice. TVD, total variation distance between transition matrix stationary distributions.

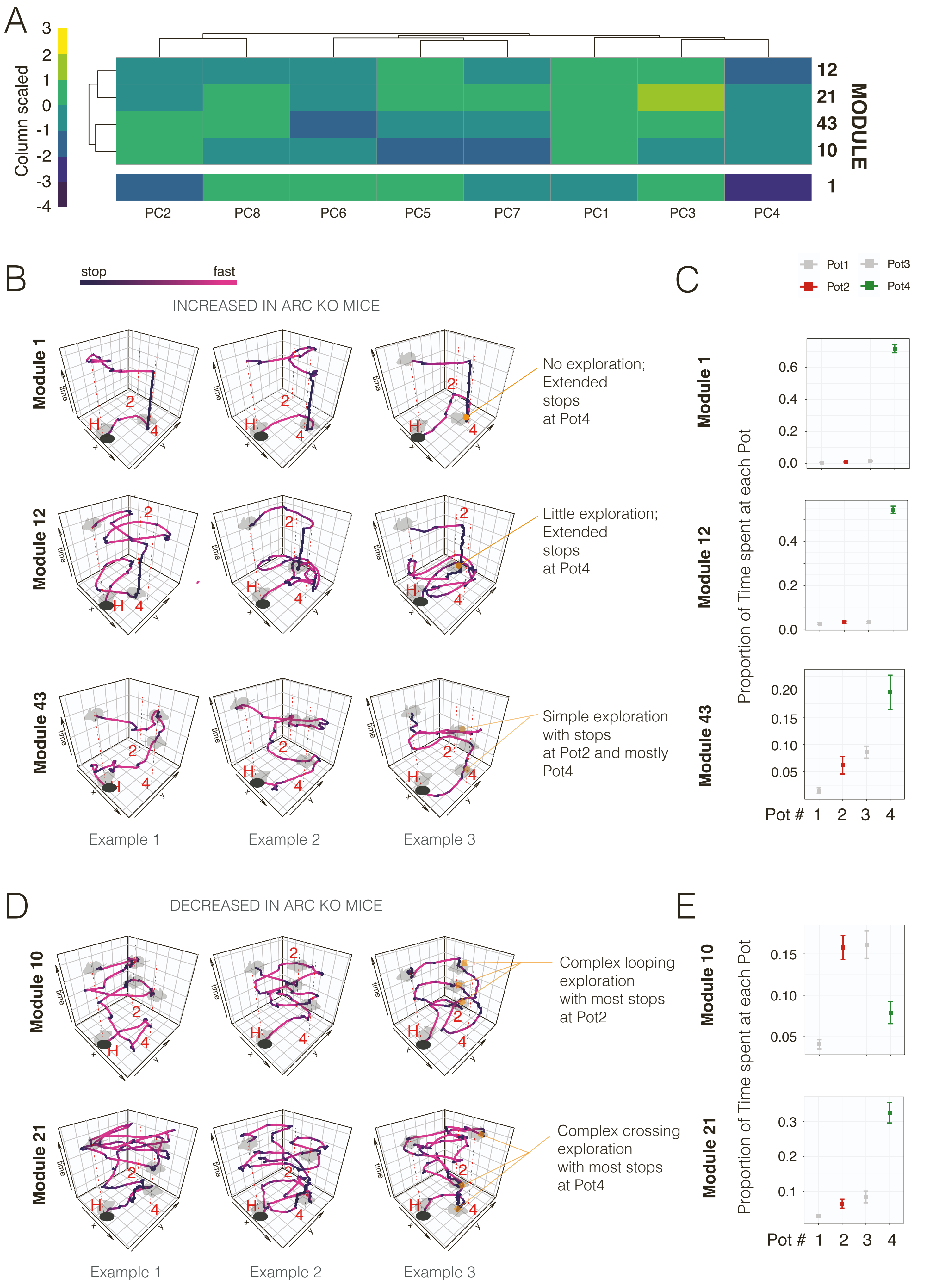

A
Column scaled
3
2
1
0
-1
-2
-3
-4
12
21
43
10
1
MODULE
PC2
PC8
PC6
PC5
PC7
PC1
PC3
PC4
B
stop
fast
INCREASED IN ARC KO MICE
Module 1
Module 12
Module 43
No exploration; Extended stops at Pot4
Little exploration; Extended stops at Pot4
Simple exploration with stops at Pot2 and mostly Pot4
Example 1
Example 2
Example 3
C
Pot1
Pot2
Pot3
Pot4
Proportion of Time spent at each Pot
Pot # 1 2 3 4
D
DECREASED IN ARC KO MICE
Module 10
Module 21
Complex looping exploration with most stops at Pot2
Complex crossing exploration with most stops at Pot4
Example 1
Example 2
Example 3
E
Proportion of Time spent at each Pot
Pot # 1 2 3 4

FIGURE S4. Characterization of Arc affected foraging modules. Related to Figure 4.

(**A**) The heatmap shows the coordinate values for each principal component from a PCA analysis of the behavior data for the centroid behavior that is representative of each shown module. The result shows how the different PCs differentiate different modules.

(**B and C**) The X-Y traces over time in (B) show three representative movement patterns from different mice for Modules 1, 12 and 43, which are modules that show significantly increased expression by *Arc*$^{-/-}$ mice. The locations of the home (H), former food patch Pot2 (2) and active food rich patch Pot4(4) are shown. Mouse stopping positions are illustrated by grey mice. Module 1 and 12 show simple decision and action sequences that are focused on the food containing patch in Pot4. Module 43 involves a simple exploration with brief stops at Pot4 and Pot2. The graphs in (C) show the average proportion of time spent at each pot for each module type across different mice. Modules 1 (n= 43 mice), Module 12 (n=44 mice) and Module 43 (n=19) show that most time is spent at Pot4. Module 43 also involves time at the former food patch, Pot2. Plots show mean $\pm$ SEM.

(**D** and **E**) The X-Y traces show representative movement patters for three different mice for Modules 10 and 21 (D), which have significantly decreased expression in *Arc* $^{-/-}$ mice compared to $^{+/+}$ littermates. Both modules are complex compound decision and action sequences that involve explorations and interactions with different elements of the environment. Module 10 has a simpler looping structure compared to Module 21. The graphs in (E) show the proportion of time spent at each pot, revealing that Module

10 (n=26) involves increased time at the former food patch (Pot2), while Module 21 involves time at Pot4 with some time at Pot2 (n=34).
